# NF-κB Inducing Kinase Regulates Intestinal Immunity and Homeostasis

**DOI:** 10.3389/fimmu.2022.895636

**Published:** 2022-06-27

**Authors:** Bingran Wang, Jun Shen

**Affiliations:** ^1^ Division of Gastroenterology and Hepatology, Key Laboratory of Gastroenterology and Hepatology, Ministry of Health, Inflammatory Bowel Disease Research Center, Renji Hospital, School of Medicine, Shanghai Institute of Digestive Disease, Shanghai Jiao Tong University, Shanghai, China; ^2^ Ottawa-Shanghai Joint School of Medicine, Shanghai Jiao Tong University School of Medicine, Shanghai, China

**Keywords:** NIK, non-canonical NF-κB, intestinal immunity, intestinal homeostasis, IBD – inflammatory bowel disease

## Abstract

Intestinal immunity and homeostasis are maintained through the regulation of cytokine trafficking, microbiota, necrosis and apoptosis. Intestinal immunity and homeostasis participate in host defenses and inflammatory responses locally or systemically through the gut-organ axis. NF-κB functions as a crucial transcription factor mediating the expression of proteins related to the immune responses. The activation of NF-κB involves two major pathways: canonical and non-canonical. The canonical pathway has been extensively studied and reviewed. Here, we present the current knowledge of NIK, a pivotal mediator of the non-canonical NF-κB pathway and its role in intestinal immunity and homeostasis. This review also discusses the novel role of NIK signaling in the pathogenesis and treatment of inflammatory bowel disease.

## 1 Introduction

The intestine is the largest component of the human immune system (1). Intestinal immunity and homeostasis are complex and sophisticatedly regulated by abundant innate and adaptive immune cells, mucous associated lymphoid tissue, and trillions of commensal microorganisms (2). Because of the complexity of intestinal immunity and its connection to the immune system, dysregulation of the intestinal immunity leads to local or systemic inflammatory responses, causing impaired absorptive function or even translocation of microbiota, and is involved in the progression of several inflammatory diseases. Disturbances in intestinal immunity and homeostasis emerge as the pathogenesis of inflammatory bowel disease (IBD) and systemic immune activation, or lead to the progression of chronic metabolic diseases, such as diabetes mellitus (3).

Nuclear factor-κB (NF-κB) is a family of transcription factors that serves to regulate inflammatory and immunological responses by controlling the expression of a large number of targeted genes in response to changes in the environment (4, 5). The activation of NF-κB is mediated by two major pathways, namely canonical and non-canonical, which mediate the signaling downstream of different receptors, have different signaling cascade component and implement different biological functions *via* activating different subtypes of NF-κB. NF-κB inducing kinase (NIK) is a crucial non-canonical mediator of the NF-κB signaling cascade, and mainly responds to signals transduced by the tumor necrosis factor receptor (TNFR) superfamily. In the past two decades, the role of the non-canonical NF-κB signaling pathway in mucosal immunity and homeostasis has been highlighted. Numerous research illustrate NIK is involved in the regulation of intestinal immunity and homeostasis through activating the development of effector T cells and IgA secretion (6–9), stimulation of the secretion of cytokines induced by microbiota (10), maintenance of microfold cell function (11), and sustaining the function of regulatory T cells (Tregs) and suppressor dendritic cells (DCs) (12, 13). Moreover, the close relationship between aberrant NIK signaling and pathogenesis of IBD has been extensively studied (14, 15). Consequently, this article reviews the signaling pathways mediated by NIK and its relevance to the pathogenesis and treatment of IBD, including ulcerative colitis (UC) and Crohn’s disease (CD).

## 2 NF-κB Is Activated by Canonical and Non-Canonical Pathways

NF-κB is a family of transcription factor, including NF-κB1 (p50/p105), NF-κB2 (p52/p100), RelA (p65), RelB, and c-Rel in mammals, and regulates the transcription of kappa chain in B cells *via* binding to κB enhancer as homodimers or heterodimers (16). NF-κB shares two common motifs: transcription activation domain (TAD) and N-terminal Rel homology domain (RHD). The former is responsible for positive regulation of gene expression by recruiting coactivators and the latter is responsible for NF-κB dimerization and DNA binding (17). The phosphorylation of TAD facilitates the recruitment of CREB binding protein (CBP)/p300 coactivator (18), which decreases the levels of histone deacetylases 3 (HADC3), resulting in the augmentation of histone acetylation and subsequent enhancement of the transcription of target genes (19).

**Figure 1:**
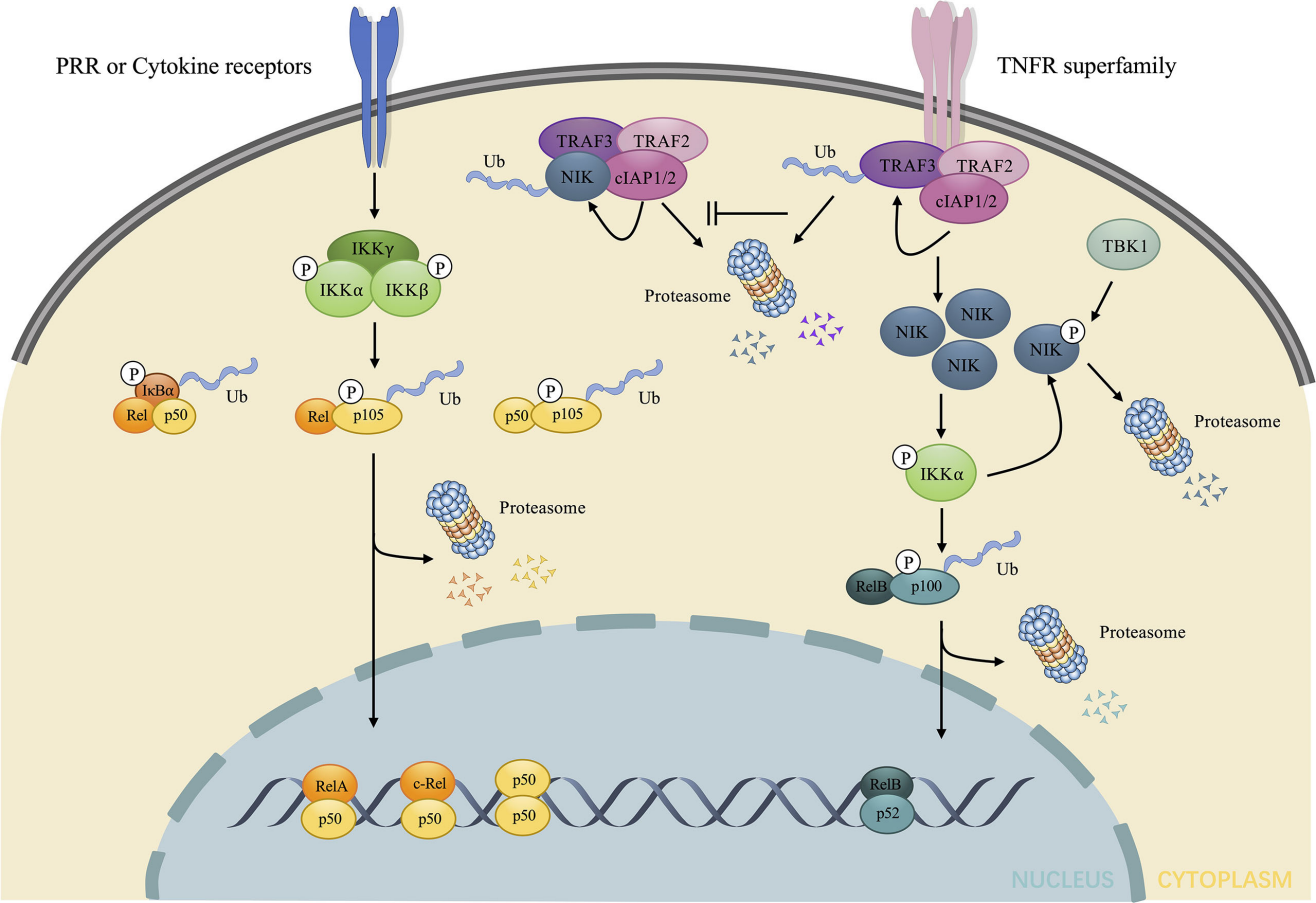
Brief illustration of the activation and regulation of the two major NF-κB signaling pathway. The canonical pathway is induced downstream of the stimulation of LPS and cytokines. Such stimuli mediate the activation of IKK, consisting of three subunits: IKKα, IKKβ, and IKKγ, which in turn phosphorylates and elicits the ubiquitin dependent processing of IκB and p105, leading to the nuclear translocation of RelA/p50, c-Rel/p50, p50/p50 dimers and the regulation of target gene expression. The non-canonical pathway is induced downstream of TNFR superfamily. In the absence of stimuli, NIK undergoes ubiquitin-dependent degradation mediated by NIK ubiquitin ligase, composed of TRAF3, TRAF2 and cIAP1/2. Under stimuli, TRAF3 undergoes ubiquitin-dependent degradation, which allows the accumulation of NIK. NIK directly phosphorylates and activates IKKα, contributing to the phosphorylation and ubiquitin dependent processing of p100 to p52. The nuclear accumulation of p52/RelB dimers changes transcriptional activity of target genes. The excessive NIK can also be evacuated *via* negative feedback mechanisms. IKKα can phosphorylate NIK, resulting in the direct proteasome-mediated degradation without the involvement of NIK ubiquitin ligase. TBK1 downstream of CD40 and BAFF also phosphorylates NIK and induces degradation. LPS, lipopolysaccharides; IKK, IκB kinase; TNFR, tumor necrosis factor receptor; NIK, NF-κB inducing kinase; TBK1, TANK-binding kinase 1; BAFF, B-cell activating factor belonging to the TNF family.

NF-κB, exerts immunoregulatory function by enhancing the transcription of target genes under specific stimulations. In the physiological state, NF-κB is sequestered in the cytoplasm and is deprived of the nuclear translocation by inhibitors of NF-κB (IκB), among which the most representative IκB is IκB. p105 and p100, as precursors of p50 and p52, respectively, also exhibit an IκB-like structure in their C-terminal portion, and perform similar functions as IκB (20). NF-κB activation is mediated by both canonical and non-canonical pathways, depending on the types of stimulus (**Figure 1**) (21).

### 2.1 Canonical Pathway

Under numerous stimuli, such as ligands for cytokine receptors, NF-κB is activated *via* the canonical pathway. IκB kinase (IKK), consists of the catalytic subunits: IKKα, IKKβ, and a regulatory subunit: IKKγ, also known as NF-κB essential modulator (NEMO) (22, 23), which plays a key role in the canonical pathway by phosphorylating IκB, inducing its ubiquitination and proteasomal degradation. This process promotes the nuclear translocation of the NF-κB dimer, predominantly p50/RelA and p50/c-Rel, which enhances the transcription of proinflammatory cytokines.

### 2.2 Non-Canonical Pathway

Under the stimulation of certain receptors, such as the lymphotoxin-β receptor (LTβR) (8), TNFR superfamily, CD40 (24), and B-cell activating factor (BAFF) receptor (BAFFR) (25), the activation of NF-κB is triggered by a non-canonical pathway. NIK, as a prototypical activator of the non-canonical pathway (26) maintains a low level without stimuli as a result of ubiquitin-dependent degradation mediated by TNF receptor-associated factor-3 (TRAF3), which provides ubiquitination substrate binding sites and complexes with TRAF2 and cellular inhibitor of apoptosis1/2 (cIAP1/2) to form NIK ubiquitin ligase (27). However, the stimulation of certain receptors leads to the degradation of TRAF3 after ubiquitylated by cIAP1/2, thus contributing to the accumulation of NIK (28). Since NIK is degraded *via* NIK ubiquitin ligase upon synthesized, the accumulation of NIK takes time for *de novo* synthesis, which explains why the non-canonical pathway is slow and dependent on protein synthesis compared to the canonical pathway (29). Subsequently, NIK activates IKKα *via* phosphorylation and complexation with IKKα and p100 (30) to enhance the phosphorylation of p100 at Ser866 and Ser870 by IKKα without the help of IKKβ and IKKγ subunit (31). The phosphorylation of p100 creates sites that are bound by TrCP and induces the ubiquitination and proteasome limited degradation, not only producing mature p52 (NF-κB2) but also initiating nuclear translocation of p52 (32). The mature p52 prefers interacting with RelB. Consequently, the predominant NF-κB dimer in the non-canonical signaling is p52/RelB.

However, upon phosphorylated by NIK, IKKα will also phosphorylate NIK at Ser809, Ser812, and Ser815 (33), which disturbs the stability of NIK, resulting in direct proteasome mediated degradation independent of cIAP1/2 and ubiquitylation (34). TANK-binding kinase 1 (TBK1), induced by anti-CD40 and BAFF, also phosphorylates NIK at Ser862, which is located in the degradation-determination region, and thus triggers the degradation of excessive NIK without the involvement of NIK ubiquitin ligase (35). Unlike TRAF-cIAP ubiquitin E3 complex mediated physiological degradation of NIK in the unstimulated state, both pathways mentioned above show a negative feedback of the NIK axis after stimulation, aimed at inhibiting excessive stimulation and thus preventing immune disorders or oncogenesis. Impaired negative feedback of the non-canonical NF-κB signaling is associated with autoimmune diseases, such as systemic lupus erythematosus (SLE), and IBD (14, 36, 37) and the sustained NIK signaling is considered as an oncogenic event in diffuse large B-cell lymphoma (38). In conclusion, NIK is the central core of the non-canonical NF-κB signaling (**Figure 1**).

## 3 NIK in Intestinal Immunity and Homeostasis

### 3.1 NIK Modulates Adaptive Immunity

**Figure 2:**
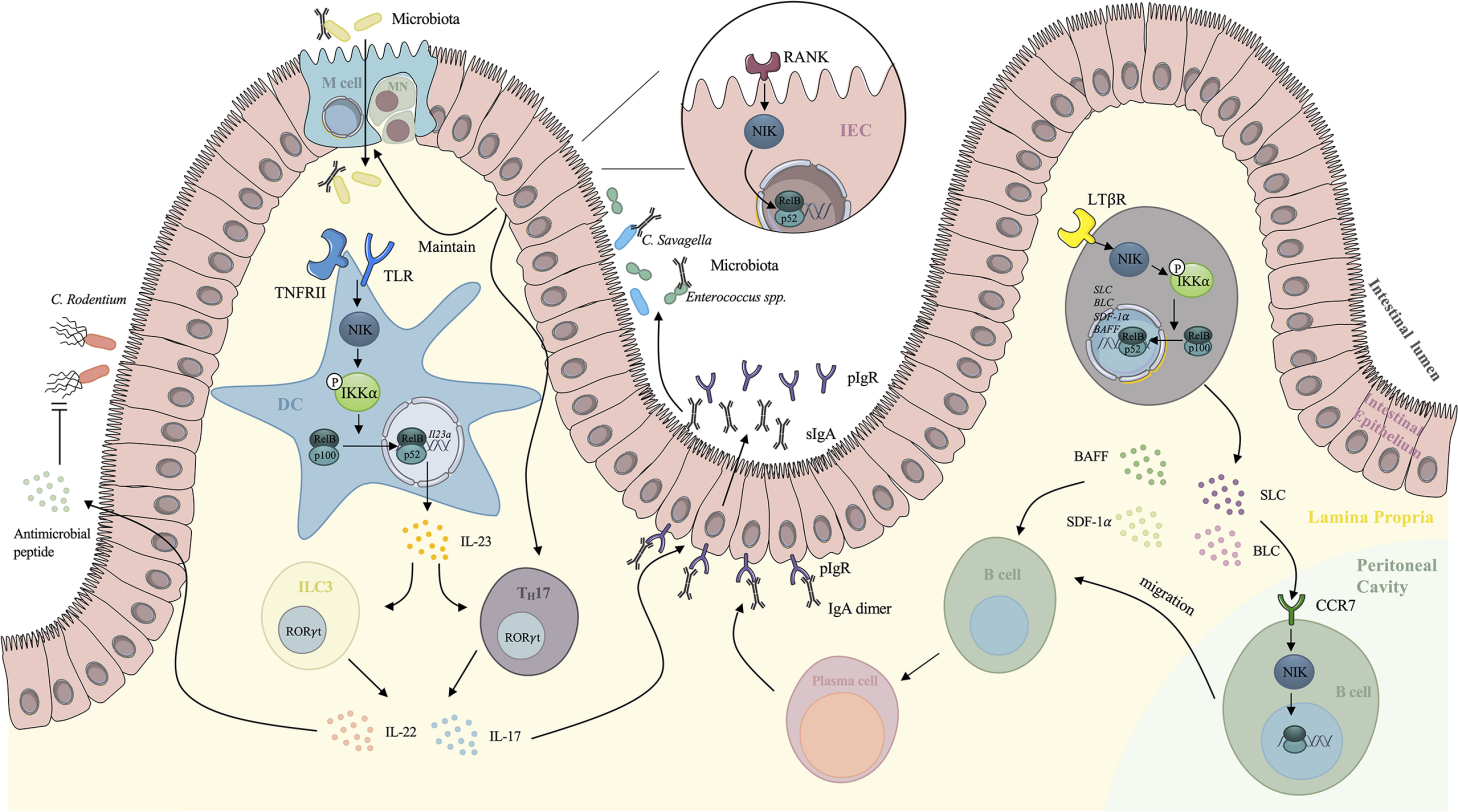
Mechanism of NIK signaling-mediated regulation of intestinal adaptive immunity. TLR, together with TNFRII induces NIK signaling in DCs. The nuclear translocation of RelB/p52 enhances the expression of IL-23, which maintains the TH17 cells and ILC3s. IL-17 secreted by TH17 cells and ILC3s stimulates the expression of pIgR on the basal surface of IECs, which increases the secretion of IgA to intestinal lumen. IL-22 secreted by TH17 cells and ILC3s also stimulates antimicrobial peptide against *Citrobacter rodentium*. NIK signaling downstream of RANK on IECs mediates the maintenance and differentiation of M cells, which facilitates the delivery of antigens to immune cells. NIK in IECs also induces IL-17 expression and IgA secretion, which enhances the intestinal immunotolerance. NIK signaling also modulates the migration of peritoneal cavity B cells to GALTs. Under the stimulation of SLC and BLC, NIK is activated in peritoneal cavity B cells and it mediates the migration. NIK participates in the downstream of LTβR in the intestinal stromal cells as well *via* upregulation of SDF-1 and BAFF, providing the microenvironment for B cells maturation. NIK also upregulates SLC and BLC, inducing B cells migration. NIK, NF-κB inducing kinase; DCs, dendritic cells; ILC3, type 3 innate lymphoid cells; IECs, intestinal epithelial cells; M cells, microfold cells; GALT, gut associated lymphoid tissue; SLC, secondary lymphoid tissue chemokine; BLC, B lymphocyte chemoattractant; BAFF, B-cell activating factor belonging to the TNF family.

NIK is activated in several immune cells under the stimuli of microbial invasion, functioning as an indispensable component of adaptive immunity. It has been reported that NIK participates in the development of T cells (39), regelation of IgA secretion (7), microfold cells (M cells) maintenance (11) and B cells migration (40, 41). The role of the NIK in intestinal adaptive immunity includes different parts (**Figure 2**).

#### 3.1.1 NIK Facilitates T Cells Development in Peyer’s Patches and Intestinal Secretory IgA Production

Peyer’s Patches (PPs), as secondary lymphoid tissues (SLTs) of the intestine, play an essential role in maintaining the microenvironment and maturation of lymphocytes. NIK is believed to be indispensable for cell mediated immunity and SLTs formation (42). The NIK signaling downstream of LTβR plays an essential role in formation of PPs. Mice with disrupted genes encoding LTβR lack PPs (43). Yilmaz et al. indicated that *relB^-/-^* and *nfkb2^-/-^* mice showed rudimentary PPs, and they further validated that the NIK/IKKα/p52-RelB axis mediates the development of PPs downstream of LTβR in intestinal stromal cells (44). Recent studies have elucidated the mechanisms by which the NIK stimulates the maturation of T lymphocytes in PPs (7). Instead of directly functioning in T lymphocytes, the NIK induced non-canonical NF-κB signaling mediates IL-23 induction in DCs (6) when Toll-like receptors (TLRs) are activated. This in turn maintains T_H_17 cells and type 3 innate lymphoid cells (ILC3s), both of which have the ability to express the transcription factor RORt and secrete IL-17 and IL-22 and finally enhance the expression of polymeric immunoglobulin receptor (pIgR) in the intestinal epithelium, leading to an increase in IgA secretion into the intestinal lumen independent of microbiota (7). Cytokines involved in the trafficking play a significant role in regulating intestinal immunity and homeostasis. IL-22 and ILC3s induced by IL-23 are thought to participate in the early phase of host defense against *Citrobacter rodentium* (*C. rodentium*). However, the overstimulation of NIK in DCs contributes to the exacerbation of colitis, as a consequence of IL-17 overexpression. The contribution of the IL-23/IL-17 axis has been recognized in the pathogenesis of IBD, as IL-17 induced by IL-23 elicits the release of proinflammatory cytokines, such as IL-6 and IL-8, releasing in myofibroblasts and epithelial cells which causes the recruitment of neutrophils and epithelial cell injury (45).

#### 3.1.2 NIK Maintains M Cell Function

Another player associated with the NIK signaling in the Peyer’s Patches is the M cell. M cells are specialized intestinal epithelial cell that mediates the internalization of dietary antigens and microbiota into the mucosa, initiating antigen-specific immune responses (46). Glycoprotein 2(GP2) expression on the apical surface of M cells helps the recognition of type 1 pilus-containing bacteria and the transcytosis of bacteria, which facilitates antigen recognition and processing by immune cells (47). IgA receptors on the apical surface of M cells also functions as receptors for IgA coated bacteria and mediate the engulfment (48). 1 integrin is also expressed in M cells, targeting *Yersinia enterocolitica* and inducing antigen internalization (49). Consequently, M cells play an essential role in regulating intestinal immunity and homeostasis and are related to inflammatory responses (50). Recent research has shown that the epithelial NIK signaling is involved in the M-cell maintenance, as *Nik^-/-^* mice exhibit loss of M cells in PPs (51). Ramakrishnan et al. demonstrated that epithelial RANKL mediates M-cell differentiation in duodenal and colon enteroids *via* the NIK signaling (11). Epithelial NIK signaling also elicits IL-17 and IgA which not only protect the intestine from colitis but also facilitate antigen uptake and processing by M-cells *via* IgA coating of commensal bacteria (11). All of these factors enhance the immunotolerance of the intestine and reduce inflammatory conditions, contributing to normal intestinal immunity and homeostasis. Moreover, increased levels of circulating IL-17 and IgA also protect against sepsis. However, prolonged stimulation of the NIK leads to chronic elevated IL-17 and IgA levels, resulting in intestinal injury as reviewed above. In addition, overexpression of NIK results in the ectopic expression of M cells, which worsens intestinal inflammation (52). Clinical data shows overstimulated NIK among patients with IBD, validating that uncontrolled stimulation of the NIK signaling mediates the intestinal inflammatory response (53). Consequently, intestinal homeostasis is maintained *via* the balanced activation of the NIK signaling.

#### 3.1.3 NIK Augments Peritoneal Cavity Cell Migration

One of the classical *in vivo* models to investigate the NIK signaling is the alymphoplasia (*aly*) mice, carrying a point mutation in the gene encoding NIK. As a result, impaired lymphocyte function and stromal compartment can be observed in *aly* mice (54). A special feature is that *aly* mice have a higher B1/B2 cell ratio in the peritoneal cavity (PEC) than normal mice, suggesting that antigen-specific B cells in the lamina propria (LP) are derived from PEC cells (55). Frequent migration between PEC cells and gut-associated lymphoid tissue (GALT) exists, as half of the IgA plasma cells of intestinal LP were proven to be derived from PEC cells (55). Evidence also showed that under the stimulation of secondary lymphoid tissue chemokine (SLC), NIK signaling mediated the cell migration from PEC to GALT in LP. Fagarasan et al. confirmed that *aly* PEC cells have a lower migration rate and reduced chemotactic activity of SLC and B lymphocyte chemoattractant (BLC) either at rest or under the stimulation of lipopolysaccharides (LPS) (41). Furthermore, NIK also mediates the migration of PEC cells downstream of the SLC receptor. The stromal cells in Peyer’s Patches are involved in the migration of PEC cells. The NIK signaling also modulates the secretion of BLC and SLC to provide microenvironment for B cells migration and class switch (56). Dejardin and colleagues reported that the activated LTβR in stromal cells can elicit the NIK/IKKα axis, resulting in the processing of p100, a precursor of p52, which leads to the nuclear translocation of p52. After dimerized with RelB, p52 upregulates SLC, BLC, stromal cell derived factor-1 (SDF1-α), and BAFF (40). SLC and BLC mediate the migration of PECs to GALT. SDF1-α and BAFF provide the microenvironment for B cell maturation (57, 58), thus facilitating the migration of B cells. Kunisawa et al. examined cytokines secretion related to peritoneal B cell trafficking in *aly* stromal cells, and confirmed that the NIK signaling in stromal cells facilitated B cell emigration from the peritoneal cavity by enhancing the expression levels of VCAM-1 and ICAM-1 on stromal cells and regulating the balance of CXCL13 expression (59).

#### 3.1.4 NIK Mediates Naïve B Cell Migration to Intestinal Lamina Propria

PPs function as secondary lymphoid tissues in the gut, where stromal cells provide microenvironment for B cells differentiation and homing. It has been proven that the majority of IgA plasma cells in LP are derived from PPs, and IgA^+^ B cells generated in PPs prefer to migrate to the intestinal LP (60). Suzuki et al. applied NIK deficient *aly* mice and showed that the NIK signaling downstream of LTβR in intestinal stromal cells mediates the naive B cells migration to intestinal LP. However, NIK signaling is not involved in the migration of B cells in PPs to LP. (61)

### 3.2 NIK Contributes to Innate Immunity

**Figure 3:**
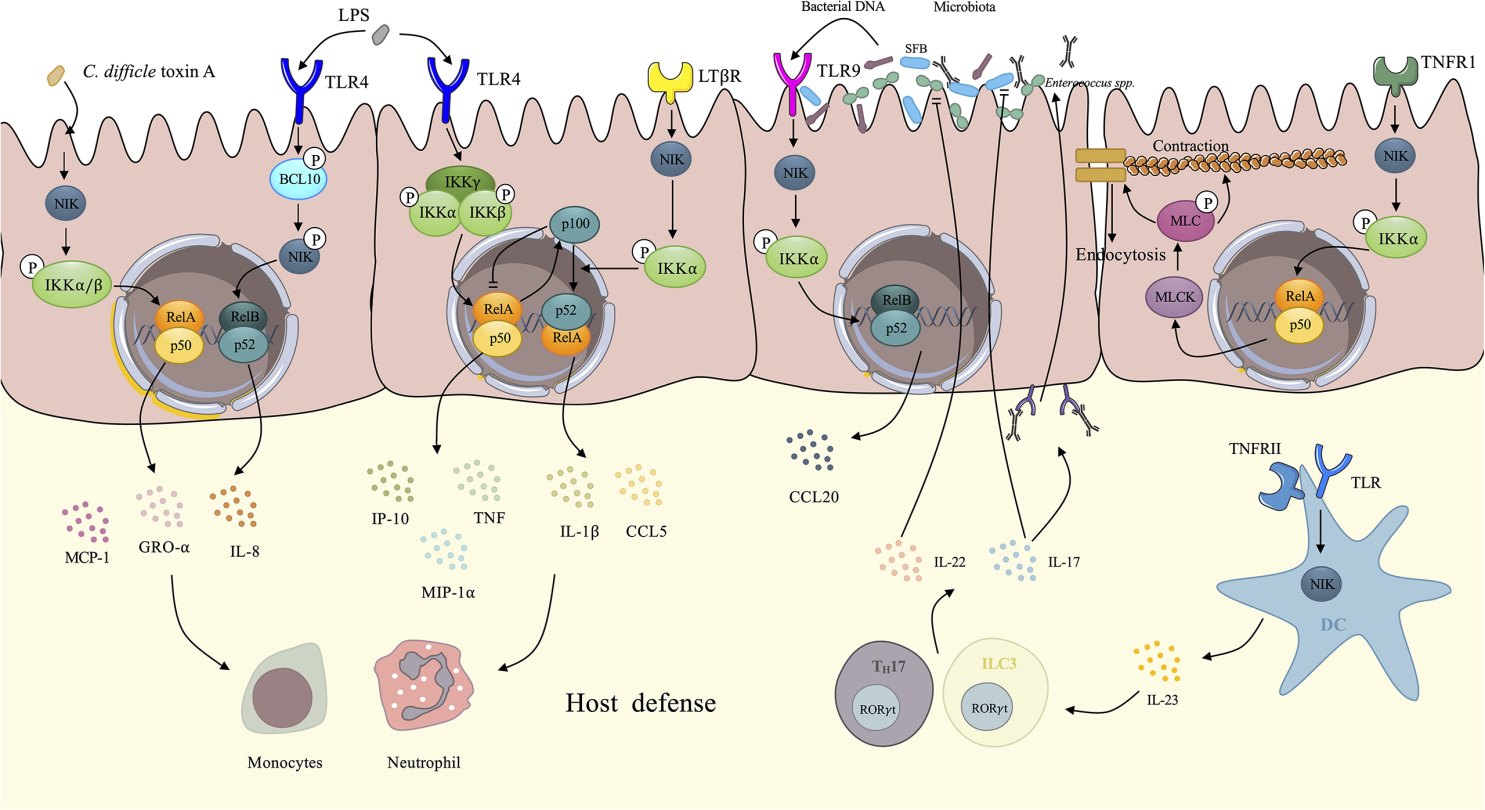
Demonstration of NIK regulates intestinal innate immunity. IECs upregulate several proinflammatory cytokines *via* NIK signaling. Upon the stimulation of *Clostridium difficile* toxin A, IECs activates the NIK/IKKα axis, resulting in the nuclear translocation of RelA/p50, which facilitates the expression of IL-8, GRO-α, and MCP-1. Under the stimulation of LPS, TLR4 on the IECs activates NIK by phosphorylation of BCL-10, which facilitates the nuclear translocation of RelB/p52 and enhances the expression of IL-8. The canonical IKK complex also mediates the signaling transduction downstream of TLR4, eliciting transcription of TNF, GRO-α, and MCP-1. In the meantime, RelA induces the expression of p100 as negative feedback to cease canonical NF-κB signaling. However, p100 can be utilized by NIK signaling downstream of LTβR, not only evacuating the inhibitory effect of p100, but also promoting the processing of p100 to p52. After dimerized with RelB, p52 upregulates IL-1 and CCL5. These proinflammatory cytokines initiates host defense *via* recruitment of neutrophils and monocytes. TNF has the ability to increase intestinal tight junction permeability through the activation of NIK/IKKα axis, which induces the nuclear translocation of RelA/p50, and activates MLCK/MLC axis, contributing to the loss of the tight junction proteins: occludin and claudin-1. Finally, the contraction of actomyosin filament leads to the opening of tight junction. Increased secretion of IgA induced by NIK signaling in DCs changes the microbiota. With IgA coating, evident downregulation of *Enterococci* and SFB can be observed. IL-22 and IL-17 induced by NIK signaling in DCs also has an inhibitory effect on *Enterococci* and SFB dysbiosis respectively. The microbiota DNA also stimulates TLR9 on IECs induces CCL20 secretion *via* NIK signaling. NIK, NF-κB inducing kinase; IECs, intestinal epithelial cells; DCs, dendritic cells; TNF, tumor necrosis factor; SFB, segmented filamentous bacteria.

The intestinal epithelium contributes to the defense against pathogen invasion, and the microbiota lying on the surface of the intestinal lumen interacts with the epithelium, both of which contribute to intestinal innate immunity. Here, we demonstrate concrete mechanisms of intestinal innate immunity driven by the NIK (**Figure 3**).

#### 3.2.1 NIK Mediates Cytokine Release in the Intestinal Epithelium

The intestinal epithelium is a single layer of cells lining on the intestinal lumen, functioning as a barrier against pathogens and a coordinating hub for immune defense events such as cytokine trafficking (62). IECs are reported to upregulate several cytokines under the stimulation of bacterial toxin. Research has also related the NIK to the bacterial toxin-induced expression of cytokines by IECs.

Kim et al. elucidated that the NIK signaling mediates *Clostridium difficile* (*C. difficile*) toxin A-induced cytokines expression by IECs (63). Upon the stimulation of *C. difficile* toxin A, phosphorylated NIK activates IKKα/β. IKKα and IKKβ then directly phosphorylate serine residues on IκB, resulting in the degradation of IκB and nuclear translocation of the NF-κB dimer, p65/p50 and p65/p65. Proinflammatory cytokines such as IL-8, growth regulated protein-α(GRO-α), and monocyte chemoattractant protein-1(MCP-1) are upregulated by transcription factor p65/p50 and p65/p65 dimers, contributing to the innate immunity of the intestinal tract (64). The engagement of non-canonical NF-κB in mediating LPS-induced immune response is well recognized (65). It has been reported that the NIK mediates intestinal innate immunity by transducing signals downstream TLR4 induced by LPS in IECs. After activation by LPS, TLR4 transduces signals by phosphorylating B cell lymphoma/leukemia (BCL)-10 at Ser138, which phosphorylates NIK without changing the level of NIK. This further leads to the activation of IKKα and nuclear translocation of the RelB-p52 dimer and subsequent induction of IL-8 secretion and inflammatory responses (66). Banoth et al. revealed that IEC LTβR induced NIK/IKKα signaling provided a co-stimulatory signaling to sustain canonical RelA/NF-κB signaling by TLR4 in response to *C. rodentium (* 67 *).* Researchers has shown that activation of the NIK/IKKα signaling downstream of LTβR not only led to a rapid augmentation of canonical NF-κB targeted pro-inflammatory genes, including TNF, interferon inducible protein-10 (IP-10), and macrophage inflammatory protein-1α (MIP-1α), but also sustained the prolonged accumulation of IL-1 and C-C chemokine ligand 5 (CCL5) downstream of TLR4 after activation by LPS (67). The canonical NF-κB component, RelA, elicits the expression of p100 as negative feedback by utilizing its inhibitory domain to terminate sustained activation (68) downstream of TLR4. However, p100 also dimerizes with RelB as a precursor of non-canonical NF-κB (69), which is utilized by the NIK signaling downstream of LTβR, preventing the inhibitory effect of p100 as well as enhancing the non-canonical NF-κB signaling and finally forming a positive loop to maintain proinflammatory responses to counter bacteria (67). In agreement with previous findings, a recent publication reported that non-canonical NF-κB signaling enhanced canonical RelA mediated inflammatory responses in IECs and exacerbated colitis (70). They also revealed an upregulated non-canonical NF-κB signaling in IBD patients, indicating that uncontrolled IEC-specific NIK signaling involves in the pathogenesis and progression of IBD (70). In physiological state, intestinal inflammatory response is self-limiting rather than fulminant due to the negative regulatory function of NIK signaling. It has been reported that IKKα induced by NIK participates in the negative regulation of inflammation *via* its kinase property (71, 72). Lawrence et al. elucidated that IKKα accelerated canonical c-Rel and RelA turnover and dissociation from promoters of pro-inflammatory genes in macrophages through direct phosphorylation (72). IKKα also negatively regulates apoptosis-associated specklike protein containing a CARD (ASC) through phosphorylating at Ser193 and Ser16 and interferes with the assembly of nod-like receptor P3 (NLRP3) inflammasome (71). These negative regulation mechanisms in NIK plays an essential role in the reduction of uncontrolled inflammation and the maintenance of intestinal homeostasis. These findings not only elucidated the correlations between the NIK signaling and IEC-mediated innate immunity, but also revealed the crosstalk between non-canonical and canonical NF-κB. (**Figure 3**). These findings seem contradictory that NIK signaling not only induces intestinal inflammation but also negatively regulates inflammatory responses. Actually, NIK plays its role in regulating intestinal homeostasis in a cell-specific way. Under the stress of pathogen invasion, NIK signaling in IECs promotes adaptive immunity against pathogens while macrophages inhibits excessive inflammation through NIK signaling to protect intestinal tissue from damage (72).

#### 3.2.2 NIK Modulates Intestinal Tight Junction

The intestinal tight junction (TJ) plays a significant role in gut immunity and homeostasis (73). Evidence has shown that paracellular permeation of intestinal antigens mediated by defective TJs can induce or propagate inflammatory responses. Intestinal TJ permeability which is considered an etiological factor in CD, is significantly increased in patients with CD (74). Studies have shown that proinflammatory cytokines [e.g., TNF-α, IL-1 (75), IFN-γ (76)] are related to increased intestinal TJ permeability. TNF- α and IL-1 modulates intestinal TJ by enhancing the expression of myosin light chain kinase (MLCK) (77, 78). TNF-α also increases epithelial cell apoptosis (79), whereas IFN-γ manipulates TJ permeability *via* micropinocytosis of tight junction proteins, such as occludin, and claudin-1 instead of inducing apoptosis (80). Clinical research has also shown that an increase in intestinal TJ permeability induced by TNF-α can be observed in patients with IBD (81), and that IL-1 is also elevated in the intestinal tissues of patients with CD (82). Recently, Al-Sadi et al. defined the NIK signaling as being involved in TNF- α modulation of intestinal TJ permeability, both *in vitro* and *in vivo* (83). Research has shown that TNF causes an increase in phosphorylated NIK, and induces the phosphorylation of IKKα at Ser176 without the involvement of IKKα. Surprisingly, NIK signaling is mediated by the canonical pathway NF-κB, p50-RelA dimer, which finally results in the activation of MLCK gene and thus increasing the TJ permeability (83). MLCK was shown to catalyze the phosphorylation of MLC, contributing to the activation of Mg^2+^–Myosin ATPase, which finally results in the contraction of the peri-junctional actomyosin filament, thus leading to the tension-induced opening of the TJ barrier and the increased permeability (78). However, IL-1-induced increase in intestinal TJ permeability is mediated by MEKK-1 pathway, rather than the NIK signaling. Recent research confirms that IL-1 induced MEKK-1/IKKα canonical NF-κB pathway, and the activation of MLCK gene, without the involvement of NIK (84). In conclusion, TNF-α induced the modulation of MCLK and thus the increased intestinal TJ permeability was mediated by NIK/IKKα axis activated NF-κB p50/RelA dimer, while IL-1 achieves the same property *via* the NIK-independent canonical pathway. These findings indicate that the selectivity of canonical or non-canonical pathways targeting NF-κB is mediated by different cytokines and ultimately achieves the same physiological process: increase in intestinal TJ permeability (**Figure 3**).

#### 3.2.3 NIK Rescues Intestinal Epithelium Injury

NIK signaling is activated upon IECs detachment to reduce apoptosis and maintain intestinal homeostasis. It has been reported that IL-1, IL-1R type II and IL-6 is upregulated upon IEC detachment (85, 86). Yan et al. revealed that IECs detachment activated NIK and increased IKKα phosphorylation, resulting in the activation and nuclear translocation of p65. The p65 activation induced by NIK signaling resulted in induction of IL-1, IL-1R type II and IL-6 and reduced caspase activation and apoptosis (87). Moreover, NIK signaling is activated to alleviate intestinal injuries by facilitating regeneration (88). Tumor progression locus-2 (Tpl2), a mitogen-activated protein kinase kinase kinase 8 (MAP3K8) activates NIK signaling through phosphorylation (89). Roulis et al. demonstrated an intestinal myofibroblast (IMF)-specific pathway mediated by Tpl2 maintained intestinal homeostasis *via* promoting epithelium regeneration under injuries (88). IMF sensed the penetrated bacteria *via* TLR4. Tpl2 interacted with p105 and mediated the phosphorylation of ERK downstream of TLR4, which enhanced the expression of prostaglandin E_2_ (PGE_2_) by inducing enzymatic activity of cyclooxygenase-2 (88). PGE_2_ plays an essential role in intestinal proliferation by sustaining Wnt signaling of stem cells, which rescued the injured intestinal epithelium (90). As a result, reduced level of Tpl2 is associated with disturbance of intestinal homeostasis and IBD pathogenesis (91).

### 3.3 NIK Maintains Intestinal Regulatory Microenvironment

Intestinal homeostasis is the result of balance of pro-inflammatory and regulatory anti-inflammatory immune cells. Consequently, the imbalance of T_H_17 cells and Tregs has been considered as the pathogenic mechanism of CD (92). The regulatory microenvironment of intestine is maintained by IECs (93), CD103^+^ suppressor migration DCs (94) and different populations of Tregs and supported by tissue-resident macrophages (95). Multiple studies have shown that NIK signaling sustains intestinal regulatory microenvironment. Previous studies confirmed that the homeostasis of peripheral Tregs is mediated by NIK signaling since overexpression of NIK increases Tregs function (12). Researchers also found that conditional depletion of *nfkb2* in Tregs led to increasing number of peripheral Tregs with impaired suppressive capacity. As the result, Tregs-specific *nfkb2^-/-^* mice displayed localized immune infiltrations in the colons with increased CD4^+^ and CD8^+^ T cells expressing IFN-γ, while additional depletion of RelB reversed the phenotype (96). Further mechanism study showed that downstream of T cell receptor (TCR), increased p100 synthesis had an inhibitory effect of RelB. However, non-canonical NF-κB signaling downstream of OX40 or GITR contributed to the processing of p100 to p52 which enhanced nuclear translocation of RelB, resulting in the impaired suppressive function of Tregs (96). *nfkb2^-/-^* mice showed massive inflammation in colons, with other tissues intact, for example lungs, kidney and liver. Depletion of *nfkb2* in T cells selectively affected the peripheral Tregs without evident effects on Tregs generated in thymus, indicating that NIK signaling has specific effect on peripheral Tregs (96). In addition to Tregs-intrinsic role, NIK is involved in the maintenance of Tregs through modulating the secretion of IL-10. Serebrennikova et al. revealed that deletion of Tpl2, a MAP3K8 which regulated NIK and IKKα by phosphorylation led to impaired IL-10 expression of macrophages and DCs *via* inhibition of mTOR/Stat3 signaling downstream of TLRs. Consequently, peripheral Foxp3^+^ inducible Tregs failed to achieve suppressive function without sufficient IL-10, and shaped a pro-inflammatory microenvironment of intestinal mucosal and accelerated intestinal inflammation and oncogenesis (97).

Several researches showed that NIK signaling mediates the suppressive function of DCs through inducing expression of a key enzyme, indoleamine 2,3-dioxygenase (IDO) (13). Non-canonical NF-κB signaling mediated by NIK and IKKα promotes the expression of IDO in DCs downstream of CD40, which promotes the differentiation of T cells with regulatory properties (98). Under sustained exposure to LPS, increased level of IDO can be found in DCs, which leads to overexpression of immunoregulatory molecules, including programmed death ligand 1 (PD-L1), PD-L2, and IL-10 and finally induces DCs with tolerogenic phenotype to alleviate tissue damage or allergic reaction caused by prolonged inflammatory response. Since the interaction between RelB and kynurenine, a metabolite of IDO, DCs favors NIK signaling rather than canonical NF-κB signaling downstream of TLR4 (99). Consequently, the involvement of NIK signaling in endotoxin tolerance is mediated by interaction with IDO signaling. Yu et al. elucidated that RelB/p52 directly bound to IDO gene promoter in myeloid-derived suppressor cells of breast cancer microenvironment mediated by NIK signaling downstream of STAT3 activation (100). That is, NIK signaling is involved in the activation of IDO, which plays a central role in the suppression of immune responses. Far deeper mechanisms of interactions between NIK and IDO in shaping the intestinal regulatory immune microenvironment should be deciphered.

NIK signaling is a crucial mediator of intestinal immune tolerance. *Nik^-/-^*, *relb^-/-^*, and *nfkb2^-/-^* mice spontaneously develop autoimmunity (101). NIK essentially modulates immune regulation in a tissue-specific way. Andreas et al. confirmed that RelB deletion in DCs contributed to accumulation of tissue Tregs and showed a protective role in autoimmune encephalomyelitis (102). However, DC-specific deletion of RelB reduced intestinal RORt^+^ Tregs leading to defective tolerance of microbiome and oral antigens (102). In addition, DC-specific deletion of TRAF6, an upstream regulator of canonical NF-κB impairs intestinal Tregs differentiation, resulting in defective tolerance and aberrant type 2 allergic response after oral antigen uptake (103).

### 3.4 NIK and Microbiota

Gut microbiota are a large group of commensal bacteria that occur in the gastrointestinal tract, do not exhibit pathogenicity, and modulate intestinal immunity and homeostasis (10). The number of commensal bacteria is ten-fold more than the cells in the human body. Microbiota interacts with innate immune system, not only by creating a protective barrier on the intestinal lumen, thus preventing adhesion of pathogenic bacteria, but also by stimulating the secretion of certain chemokines or cytokines to maintain intestinal immunity (104). Currently, NIK signaling is associated with the cytokine secretion induced by commensal bacteria. Dutta et al. reported that both commensal and pathogenic bacterial DNA-induced CCL20 secretion by TLR9 in colonic epithelial cells is mediated by both NIK/IKKα/p100 (NF-κB2) phosphorylation and the MEK/ERK pathway (105). However, there was a temporal difference between these two pathways. The MEK/ERK signaling axis induces the transcription factor AP1 (cjun/cfos), which predominantly participates in the early stage of CCL20 gene transcription, while the NIK/IKKα signaling axis induces non-canonical NF-κB2 (p52/RelB), which is predominantly involved in the late phase of CCL20 expression downstream of TLR9 stimulated by bacterial DNA. CCL20 is a chemokine with antimicrobial effects against *Staphylococcus aureus (S. aureus)* and *Escherichia* (*E. coli) (* 106) and modulates the trafficking of several immune cells such as DCs, T cells, and B cells by stimulating C-C chemokine receptor (CCR) 6 (107). Jie et al. demonstrated altered intestinal microbiota, with significantly elevated *Enterococci* and segmented filamentous bacteria (SFB), for example, *C. savagella* in mice with DC specific depletion of NIK (7). The overexpression of *Enterococci* leads to opportunistic infection (108), and SFB stimulates immune cell activation and differentiation, which may exacerbate autoimmune responses (109). It was further illustrated that the IL-23/IL-17, IL-22/IgA axis induced by the NIK signaling pathway in DCs contributes to the decreased abundance of *Enterococci* and SFB, in agreement with recent research showing that IL-22 derived from ILCs inhibits *Enterococci* expansion (110). IL-17 regulates the abundance of SFB, which maintains the intestinal homeostasis and protects the intestine from inflammatory responses (**Figure 3**) (111). NIK participates in the induction of secretory IgA in both DCs and intestinal epithelial cells downstream of TLR and RANK, respectively (7, 11). Secretary IgA provides a coating for microbiota and prevents the accumulation of opportunistic pathogenic microbes under certain conditions, thus exhibiting a protective role in infection and inflammation (112). Consequently, the loss of NIK is related to increased susceptibility to colitis (11). However, sustained activation of NIK leads to high IgA coating bacteria which causes a shift to colitogenic dysbiosis and exacerbate inflammatory responses in patients with UC and CD (112). Increased level of IgA levels lead to IgA-coated microbiota enrichment, which promote the T_H_17 dependent local or systemic inflammation, worsening peripheral spondylarthritis, an extraintestinal manifestation of CD (113).

### 3.5 NF-κB Independent Roles for NIK in Intestinal Homeostasis

Several mechanisms has also been reported that NIK signaling exerts its function in maintaining intestinal homeostasis without the involvement of NF-κB. As reviewed above, IKKα, a kinase induced by NIK interferes with NLRP3 inflammasome assembly by phosphorylating ASC rather than activating RelB and p52, thereby influencing intestinal homeostasis and immunity (71). It is reported that NIK phosphorylated receptor-interacting protein kinase 1 (RIP1) and induced tissue destruction downstream of TNFR1 independent of NF-κB (114). Jung et al. revealed that overstimulated NIK induced the fission of mitochondria and cell invasion by regulation of DRP1 phosphorylation in the absence of IKKα and NF-κB, which emerges as a novel mechanism of oncogenesis (115). Upregulated NIK signaling is also observed in IBD patients (70). Combined together, these studies provide us with a possible mechanism of inflammation-cancer transition in IBD patients.

## 4 Role of NIK Signaling in the Pathogenesis of IBD

We have reviewed the role of NIK signaling in intestinal immunity and homeostasis. In this part, we will summarize mechanisms of aberrant NIK signaling in the pathogenesis of IBD through breaking intestinal homeostasis. On the one hand, upregulated non-canonical NF-κB signaling is observed in IBD patients (70). Overstimulated NIK signaling in DCs and M cells leads to increased IL-17 secretion which plays a central role in intestinal injury and exacerbate colitis (7, 11). Sustained activation of NIK also shapes microbiome containing high IgA coating bacteria which causes a shift to colitogenic dysbiosis and exacerbate inflammatory responses in patients with UC and CD (112). Non-canonical NF-κB signaling is reported to enhance canonical RelA mediated inflammatory responses in IECs and exacerbated colitis *via* crosstalk mechanisms (70). On the other hand, NIK signaling participates in the maintenance of regulatory microenvironment and intestinal tissue repair. As a result, lack of NIK signaling also contributes to the pathogenesis of IBD as well. Consequently, it has been shown that Tpl2, as an activator of NIK has significantly reduced level in IBD patients (91).

## 5 Perspectives

Currently, NIK, as the pivotal component of the non-canonical NF-κB pathway, integrates significant physiological event such as cytokine trafficking, survival signaling, and apoptosis, and has been confirmed to maintain intestinal innate and adaptive immune response of the intestinal tract. Depletion of NIK leads to impaired PP and inadequate T cell development (44). However, overstimulation of the NIK signaling pathway also contributes to the disturbance of homeostasis, leading to prolonged inflammation, which is associated with IBD pathogenesis (11). In addition, the pathogenesis and progression of several autoimmune disease are also related to NIK overexpression (116, 117). NIK inhibitors have also been designed as novel therapy for SLE (118). It is the balanced activation of NIK that maintains intestinal immunity and homeostasis. Consequently, optimizing NIK to within the normal range is a promising area for pharmacological discovery.

There are also questions that remain to be resolved regarding the NIK signaling pathway. The upregulation of the non-canonical NF-κB pathway has been reported in patients with IBD treated with anti-TNF antibody, which is associated with regression of drug efficacy (53). Patients resistant to anti-TNF treatment showed a significant upregulation of NIK and downregulation of NLRP12, a negative regulator of both canonical and non-canonical NF-κB pathways (53). Consequently, whether recent targeted therapies for IBD such as TNF-α antibody, have an effect on the NIK signaling, how the NIK signaling pathway mediates the resistance in the novel medication of IBD, and what could be the salvage of resistance to targeted therapy of IBD become major problems to be resolved.

The canonical NF-κB pathway is overstressed in mediating the response to anti-TNF therapies such as infliximab and adalimumab (119). However, an aberrant non-canonical pathway was demonstrated in patients resistant to anti-TNF treatment (53), combined with what we reviewed above, suggesting the involvement of NIK in drug resistance. Consequently, resistance to drug therapy may be mediated by such crosstalk mechanisms. Savinova et al. reported a crosstalk mechanism between the canonical and non-canonical pathways mediated by NIK (68). Sustained canonical NF-κB signaling activation leads to negative feedback that RelA induces the production of p100, which contains an inhibitory domain of RelA and functions as an IκB protein (120). However, p100 also functions as the precursor of non-canonical NF-κB, and is further processed to p52 mediated by NIK. Consequently, cooling of the canonical NF-κB pathway is possible to reverse downregulation through the NIK signaling *via* crosstalk mechanisms. The crosstalk mechanism also reminds investigators to use NIK inhibitors to reverse anti-TNF therapy resistance. Unfortunately, the crosstalk mechanism between the two pathways has not yet been fully understood and requires further investigation.

## 6 Conclusion

NIK is the central core of non-canonical NF-κB signaling and participates in the intestinal immunity and homeostasis. Both excessive and impaired NIK signaling cause the disturbance of intestinal immunity and homeostasis. NIK signaling regulates intestinal homeostasis in a spatiotemporal heterogeneous way. NIK signaling in different tissues and cells plays different roles even contradictory roles. Different activation pattern of NIK signaling can also be seen at early and late phase response to pathogens. Advanced knowledge of the NIK in the regulation of intestinal immunity and homeostasis can provide new perspectives on inflammatory diseases. NIK is closely related to the pathogenesis and drug resistance of IBD, and is considered as a novel target for treatment of IBD and carcinoma in gastrointestinal tract. Consequently, more sophisticated molecular mechanisms of NIK signaling in IBD are urgently needed.

## Author Contributions

BW drafted the article and JS revised it critically for intellectual content. All authors read and approved the final manuscript.

## Funding

Supported by grants from Shanghai Science and Technology Innovation Initiative (21SQBS02302), Cultivated Funding for Clinical Research Innovation, Renji Hospital, School of Medicine, Shanghai Jiaotong University (RJPY-LX-004) and National Natural Science Foundation of China (No. 81770545).

## Conflict of Interest

The authors declare that the research was conducted in the absence of any commercial or financial relationships that could be construed as a potential conflict of interest.

## Publisher’s Note

All claims expressed in this article are solely those of the authors and do not necessarily represent those of their affiliated organizations, or those of the publisher, the editors and the reviewers. Any product that may be evaluated in this article, or claim that may be made by its manufacturer, is not guaranteed or endorsed by the publisher.

## Abbreviations

IBD, Inflammatory bowel disease; UC, Ulcerative colitis; CD, Crohn’s disease; NF-κB, Nuclear factor-κB; NIK, NF-κB inducing kinase; IκB, Inhibitors of NF-κB; IKK, IκB kinase; NEMO, NF-κB essential modulator; LTβR, Lymphotoxin-β receptor; TNF, Tumor necrosis factor; TNFR, Tumor necrosis factor receptor; TAD, Transcription activation domain; RHD, Rel homology domain; CBP, CREB binding protein; HADC3, Histone deacetylases 3; TRAF, TNF receptor-associated factor; cIAP1/2, Cellular inhibitor of apoptosis1/2; TBK1, TANK-binding kinase 1; M cells, Microfold cells; DCs, Dendritic cells; TLR, Toll-like receptor; ILC3, Type 3 innate lymphoid cells; IECs, Intestinal epithelial cells; pIgR, Polymeric immunoglobulin receptor; GP2, Glycoprotein 2; *aly*, Alymphoplasia; PEC, Peritoneal cavity; LP, Lamina propria; GALT, Gut associated lymphoid tissue; SLC, Secondary lymphoid tissue chemokine; BLC, B lymphocyte chemoattractant; BAFF, B-cell activating factor; LPS, Lipopolysaccharide; SDF1-α Stromal cell derived factor-1α; GRO-α, Growth regulated protein-α; MCP-1, Monocyte chemoattractant protein-1; BCL, B cell lymphoma/leukemia; MIP-1α, Macrophage inflammatory protein-1α; CCL, C-C chemokine ligand; ASC, Apoptosis-associated specklike protein containing a CARD; NLRP3, Nod-like receptor P3; TJ, Tight junction; Tpl2, Tumor progression locus-2; MAP3K8, Mitogen-activated protein kinase kinase kinase 8; IMF, Intestinal myofibroblast; PGE_2,_ Prostaglandin E_2;_ Tregs, Regulatory T cells; TCR, T cell receptor; IDO, Indoleamine 2,3-dioxygenase; PD-L1, Programmed death ligand 1; MLCK, Myosin light chain kinase; CCR, C-C chemokine receptor; RIP1, Receptor-interacting protein kinase 1.
